# Supplementary material for: Glycyrrhizic Acid Prevents Paclitaxel-Induced Neuropathy via Inhibition of OATP-Mediated Neuronal Uptake
Source: Cells. 2023 Apr 25;12(9):1249. doi: 10.3390/cells12091249 (PMC10177491; doi:10.3390/cells12091249)
Supplement: Supplementary file 1 [file cells-12-01249-s001.zip › cells-2281674-supplementary.pdf]

Supplementary Materials

Table S1. Primers for drug transporter overexpression.

Target	Primer sequence
OATP1A1	CAAAGGCCTCTGAGGCCGAAACATTTGAAGAACACCATGG<br>GTTGCGGCCGCCAGCTTAGTTTTCAGTTCTCC
OATP1A2	ACAGCTAGCATGGGAGAAACTGAGAAAAGAATTG<br>GTTGCGGCCGCTTACAATTTAGTTTTCAATTCATC
OATP1B1	CAAAGGCCTCTGAGGCCATCTATATTTCAATCATGGAC<br>GTTGCGGCCGCACAATGTGTTTCACTATCTGCC
OATP1B2	AAAAAGCTTTCACAACCACTGTTCAATCATGG<br>GTTGCGGCCGCAAGAGGTGTTTCACTGTTTTTTTC
OATP2A1	AAAAAGCTTGCCAGCCGCCCGCAGCCATGGGG<br>GTTGCGGCCGCGATGAGGCCTGCCGCCTTCTG

Table S2. CRISPR/Cas9 sequences.

Target	Sequence
OATP1A1	GCGCCAAAGTAAACAGGTGCCGG
	AGTTCAGCACCCTCTATATGGGG
OATP1B2	GCTAAGCTATACGTAGACGTTGG
	GATTTGCATCCTGCCAGACAAGG

Table S3. Primers for drug transporters.

Primer	Primer sequence	Product length (bp)	Melting temperature (°C)
OATP1A1-fw	GTGACCCCCACACTACACTT	179	59.24
OATP1A1-rv	CAGCTCTAAATACTTCCAACTGTGA		58.83
OATP1A4-fw	CAAACTCTTGCAAGTTGCCC	138	58.14
OATP1A4-rv	TTCCAGTTTTATAGCAAAAGTCAGT		57.18
OATP1A5-fw	TGATGTGGATGGAACTAACAATGAC	163	59.35
OATP1A5-rv	TGCATTTATCTGGAGCACACTTG		59.56
OATP1B2-fw	AATGACATCACCCACTGGACC	203	52.38
OATP1B2-rv	TTCGGACACTGTCTAGGTGC		55.00
OATP2A1-fw	CCGGGCCTGAGAGTTTAGTT	156	59.39
OATP2A1-rv	CAGAAGGCAAGACTCCCTCT		58.73
OATP2B1-fw	ACGACTTTGCCCACCATAGC	117	60.68
OATP2B1-rv	CCACGTAAAGGCGTAGCATGA		60.74

Table S4. Primers for neurotoxicity markers.

Primer	Primer sequence	Product length (bp)	Melting temperature (°C)
ATM-fw	TCACCTTAAGGGTTCTCGTCG	147	59.46
ATM-rv	TTCGAACTCTCGAGGCTGTG		59.76
BAD-fw	TGGCTATGTTCCCTCTCCGT	124	60.33
BAD-rv	CACGTTTCTTGACCTGGGGC		61.52
BAX-fw	AGGACGCATCCACCAAGAAG	166	60.04
BAX-rv	CAGTTGAAGTTGCCGTCTGC		60.04
BCL2-fw	TCTTTGAGTTCGGTGGGGTC	105	59.60
BCL2-rv	GATGCCGGTTCAGGTACTCA		59.46
BDNF-fw	TACCTGGATGCCGCAAACAT	182	60.03
BDNF-rv	TGGCCTTTTGATACCGGGAC		60.03
DAPK1-fw	ACACCTCCCTCTACCCAGTC	108	59.96
DAPK1-rv	GCTTCCCAAGTGACCGAAGT		60.25
Gpr37-fw	CAGGACAACTCCAGGGTTCC	106	59.96
Gpr37-rv	CAGTCATCTGCAGACCCGTT		60.04
Gsn-fw	CGTCACCATGGCTCCGTATT	142	60.18
Gsn-rv	CGTCTCAGACACCCGACTTT		59.69
LTA-fw	CGGTAACAGGCATCCCTCAG	105	60.18
LTA-rv	GGTGTCATGGGGAGAACCAG		60.04
nfkb1-fw	AGTCCCGCCCCTTCTAAAAC	118	59.67
nfkb1-rv	CCTGGATCACTTCAATGGCCT		60.06
Usp7-fw	CCGAGGACATGGAGATGGAAG	160	59.93
Usp7-rv	AACTGAAAGGTTGCCTCGGA		59.53

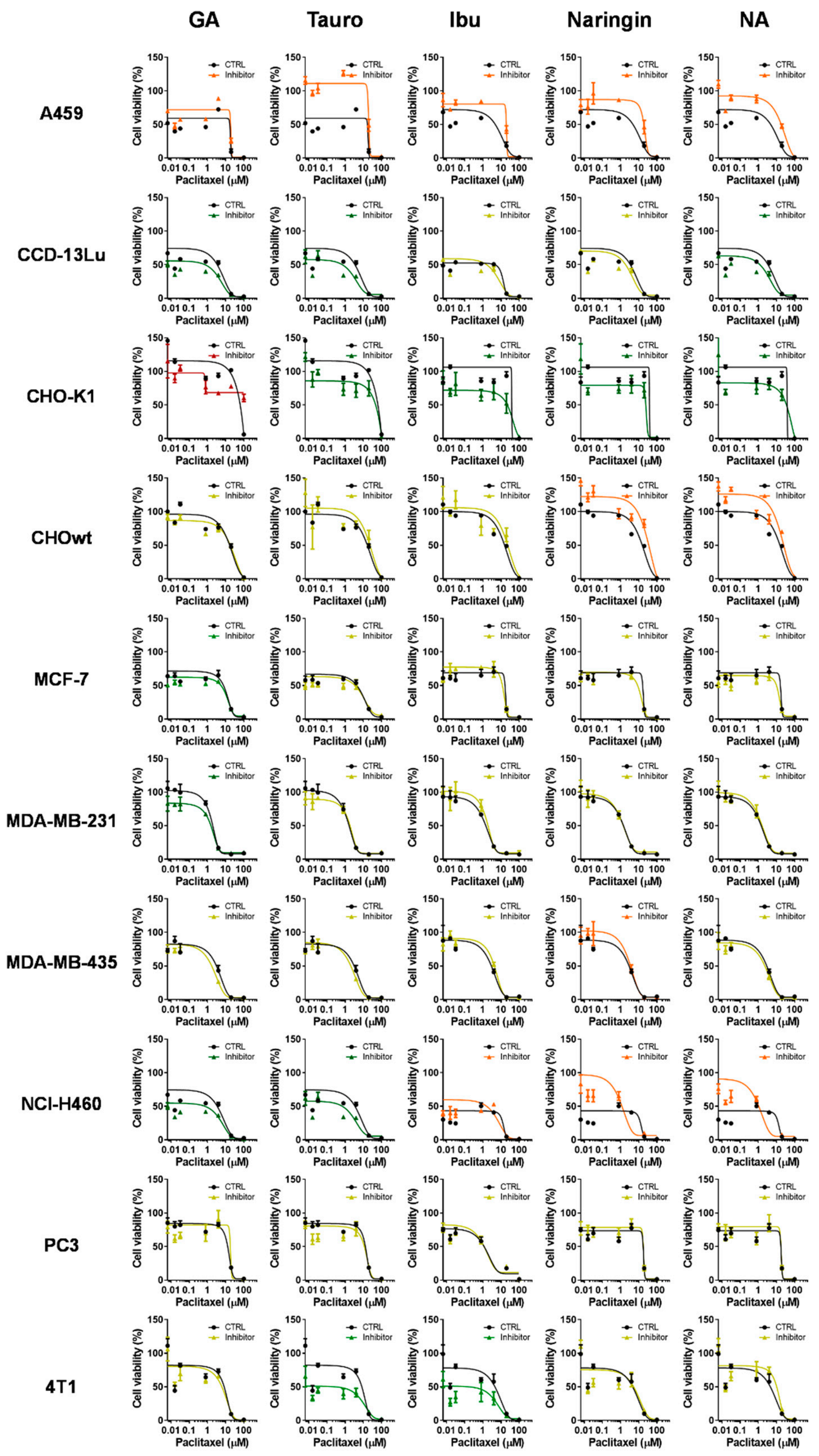

Figure S1.: Corresponding data to figure 7. Cancer cell lines A459, CCD-13Lu, CHO-K1, CHOwt MCF-7, MDA-MB-231, MDA-MB-435, NCI-H460, PC3, and 4T1 were treated with inhibitors glycyrrhizic acid (GA), taurocholate (Tauro), ibuprofen (Ibu), naringin, and niflumic acid (NA) plus increasing concentrations of paclitaxel for 72 h. Cell viability was compared to paclitaxel treated cells and results were indicated in the corresponding color.
